# Guided growth: Current concepts and novel techniques for harnessing a child’s growth potential

**DOI:** 10.1177/18632521261456090

**Published:** 2026-06-12

**Authors:** Merel C. R. Roelen, Christiaan J. A. van Bergen, Mark F. Siemensma, Deborah M. Eastwood, Ignacio Sanpera, Jaap J. Tolk

**Affiliations:** 1 Department of Orthopedic Surgery and Sports Medicine, Sophia Children’s Hospital, Erasmus MC, Rotterdam, the Netherlands; 2 Department of Orthopedic Surgery, Amphia Ziekenhuis, Breda, the Netherlands; 3 The Catterall Unit, Royal National Orthopaedic Hospital NHS Trust, London, UK; 4 Department of Orthopaedic Surgery, Great Ormond Street Hospital NHS Trust, London, UK; 5 Division of Surgery and Interventional Science, University College London, London, UK; 6 Paediatric Orthopaedic Department, Hospital Universitari Son Espases, Palma, Spain

**Keywords:** guided growth, deformities, upper extremity, lower extremity

## Abstract

The principle of guided growth is grounded in the observations of Hueter and Volkmann, who proposed that longitudinal bone growth is stimulated by tension and inhibited by compression. The importance of guided growth lies in its ability to address limb malalignment in a more physiological and less invasive manner compared to traditional osteotomies. By modulating growth at the physis, guided growth allows for gradual correction of deformities, minimizing the disruption to the child’s developing musculoskeletal system and harnessing the child’s own growth potential. Guided growth has been most frequently applied for coronal plane angular deformities around the knee, but is increasingly used for deformities in other locations throughout the growing skeleton. A wide range of deformities with underlying idiopathic and non-idiopathic etiologies are amendable to guided growth procedures. Understanding the influence of the underlying etiology, type, and severity of deformity, the growth rate of the treated physis, and skeletal age is essential for treatment success. This review explores current concepts and novel applications of guided growth and highlights areas that warrant further investigation. The focus is on the clinical aspects of guided growth to correct angular or rotational deformities in both the upper and lower extremities.

## Key concepts

- Guided growth is useful for deformities in both upper and lower extremities
- Understanding the influence of underlying etiology, severity of deformity, growth rate of the treated physis, and skeletal age is essential for timing guided growth
- Guided growth for rotational deformities is promising but warrants further research

## Introduction

The principle of guided growth is grounded in the fundamental observations of Hueter and Volkmann, who proposed that longitudinal bone growth is stimulated by tension and inhibited by compression.^1–4^ Applying compression selectively to one side of the growth plate, while allowing the opposite side to expand freely, can progressively correct the deformity.^5,6^ This minimally invasive approach has transformed the treatment of pediatric orthopedic deformities, offering a less aggressive alternative to osteotomies, particularly for angular limb deformities.^7^

The historical roots of guided growth can be traced back to the early 20th century, when Phemister recognized the potential of physeal manipulation, using bone blocks to influence lower-limb alignment.^8,9^ However, it is Haas who laid down the principles of growth manipulation for angular deformities and delaying growth.^10^ Blount further refined these concepts, introducing staples that laid the groundwork for modern guided growth procedures.^9,11^ Métaizeau’s development of the percutaneous epiphysiodesis using transphyseal screws (PETS) and Stevens’ tension band plate (TBP) marked a significant advancement, providing a stable and reliable means of applying controlled forces across the physis.^9,12,13^ These techniques create an intra- or extraphyseal fulcrum on the side of the deformity, enabling gradual correction of deformities while minimizing the risk of physeal damage.^12^

The importance of guided growth lies in its ability to address limb malalignment in a more physiological and less invasive manner compared to traditional osteotomies.^14,15^ By modulating growth at the physis, guided growth allows for gradual correction of deformities, minimizing the disruption to the child’s developing musculoskeletal system and harnessing the child’s own growth potential.^16–18^ This approach offers several advantages, including reduced surgical morbidity, shorter recovery times, and a lower risk of complications associated with osteotomies.^19,20^

However, guided growth primarily corrects deformities at the physis even if the deformity is not at the physis. In such cases, the overall alignment can be corrected through guided growth, but one must consider the potential for secondary deformities. Studies have shown some improvement in diaphyseal deformities following guided growth, particularly in younger patients, but this is not always observed.^21^ Therefore, guided growth is not universally effective for all angular deformities.

Another important consideration is the wide range of underlying etiologies that can contribute to limb malalignment and deformities. These etiologies can be idiopathic or non-idiopathic, and the outcomes and rates of correction can be difficult to predict. In some cases, deformities arise from a normal, healthy physis that is well amendable to guided growth. In other cases, deformities arise from a pathological physis, where growth is impaired due to the underlying etiology. This often results in slower correction, longer treatment times, or unsuccessful guided growth. However, when the underlying disease can be effectively treated, growth may revert to a more normal rate, allowing for faster correction of the deformity.^21,22^

This review explores the current concepts and novel applications of this technique and highlights areas warranting further investigation. Our focus will be on the clinical aspects of guided growth for correcting angular or rotational deformities in both the upper and lower extremities.

## Mechanism of guided growth

**Figure 1.**
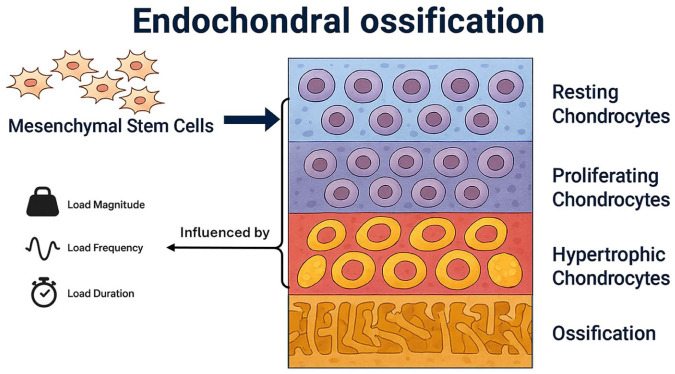
Process of endochondral ossification in long bone growth.

According to the Hueter–Volkmann principle, growth is inhibited by compression and accelerated by tension.^2^ Understanding guided growth first requires an understanding of physiological physeal growth, which occurs through endochondral ossification. In this process, mesenchymal stem cells differentiate into chondrocytes that progress through the reserve, proliferative, and hypertrophic zones before being replaced by bone.^23–25^ These zones are mechanosensitive, responding dynamically to the magnitude, frequency, and duration of applied loads. Among them, the hypertrophic zone, which is the least rigid and most deformable region of the growth plate, is the primary contributor to longitudinal growth and the most sensitive to loading differences (Figure 1).^23,26^

**Figure 2.**
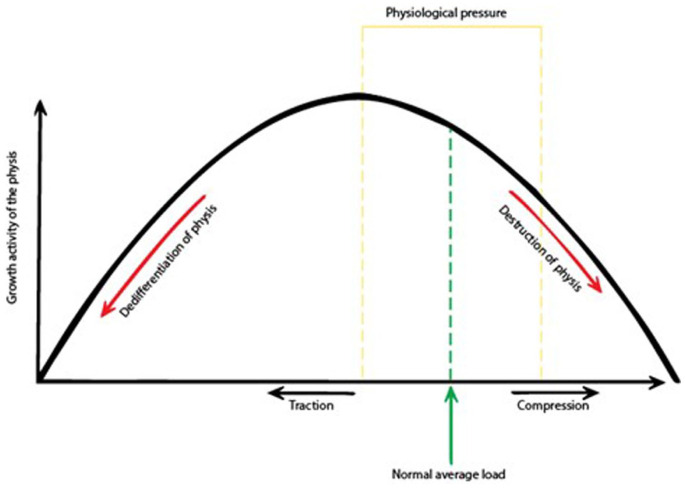
Heřt’s curve demonstrating the relationship between the load on the physis and its growth activity. Source: Adapted from Hert.

Several studies have shown that increasing the magnitude, frequency, or duration of compression progressively disrupts growth plate architecture and suppresses hypertrophic volume expansion. By contrast, dynamic tension enhances growth plate height and proliferation, particularly at moderate frequencies, whereas excessive tensile loading inhibits growth and promotes apoptosis (Figure 2).^6,23^

An important aspect of physiological growth is a negative feedback loop that corrects minor deviations in growth. By contrast, pathological physeal growth and/or abnormal mechanical forces interfere with this feedback loop, limiting its ability to correct larger deviations, leading to more persistent deformities. These deviations may exceed the capacity of correction using the feedback loop, necessitating external interventions to restore normal growth.^27,28^

Minimally invasive techniques, such as tension-band plating or PETS, utilize controlled, localized tethering to modulate asymmetric physeal growth and correct deformities. Sustained compression of approximately 0.6 MPa can completely arrest growth, while surgically applied PETS or tension-band plates generate local stresses approaching 1 MPa, sufficient to halt physeal expansion on the compressed side.^23^ Upon removal of these compression forces, growth potential is re-established, and new growth has been demonstrated.^23,29,30^ Despite this, theoretically, prolonged temporary epiphysiodesis using implants could lead to permanent growth arrest. Therefore, a practical recommendation is not to maintain implants in situ for much more than 2 years if further growth is required in the part of the growth plate following deformity correction.^6^ Knowledge of these stress thresholds and cellular responses allows optimization of implant design, placement, and timing to balance effective correction with preservation of growth potential.

In summary, guided growth is a mechanobiological process that links molecular, cellular, and tissue-level responses to mechanical stress. Controlled manipulation of these processes forms the foundation of modern strategies for correcting pediatric skeletal deformities.

## Methods

For this current concept review, a thorough search of the literature was performed through PubMed and EMBASE to identify original articles. Appropriate search terms, including “guided growth,” “hemi-epiphysiodesis,” and relevant synonyms were applied. Cross-reference search results of included studies and gray literature were included. We primarily concentrated on studies published from 2007 onward, following the introduction of the TBP, though earlier relevant studies were also considered. Most studies that could be included for this narrative review had a level of evidence of three, with a higher level of evidence found to be scarce.

We prioritized studies that investigated guided growth interventions targeting either the upper or lower extremity. Only articles published in English were considered for inclusion. Our analysis specifically focused on literature addressing angular and/or rotational deformity correction. Studies evaluating guided growth for longitudinal correction (e.g. for the treatment of leg length discrepancies) were excluded from this review.

## Guided growth in the lower extremity

Guided growth techniques have found extensive application in the lower extremity, addressing a variety of conditions affecting the hip, knee, ankle, and foot, which disrupt normal limb alignment and hence function.

## Hip

Guided growth techniques at the hip have been explored as a strategy to address hip (sub)luxation and coxa valga. Their use has been reported in several conditions, including cerebral palsy, developmental dysplasia of the hip (DDH), and hereditary multiple exostoses around the hip.^31–37^ Multiple studies have specifically examined guided growth of the proximal femur, in which a screw is placed across the medial aspect of the proximal femoral physis.^33–35,38–42^ This creates a temporary medial tether while preserving superolateral growth, resulting in progressive varus of the proximal femur.

**Figure 3.**
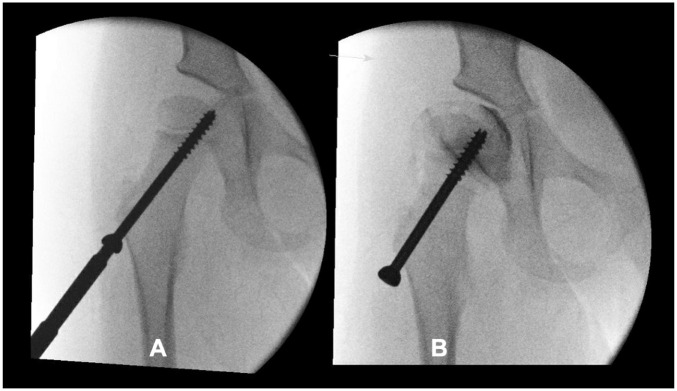
Intraoperative imaging prior to arthrogram (a). Intraoperative view with arthrogram (b). The arthrography confirms that the tip of the transphyseal screw is well within the margins of the cartilaginous femoral head.

Screw sizes of 4.5 mm or 6.5–7.0 mm have been recommended and should be tailored to patient size.^31,33–35,39^ Both partially and fully threaded screws have been used: fully threaded implants might be easier to remove and/or exchange. At least two threads should be placed in the epiphysis, but more epiphyseal purchase is beneficial. In younger children, an intraoperative arthrogram (Figure 3) is helpful for optimizing screw positioning and avoiding penetration of the joint surface.^39^ Over time, the femoral head can grow off the screw, and this may lead to the need for screw exchange.

**Figure 4.**
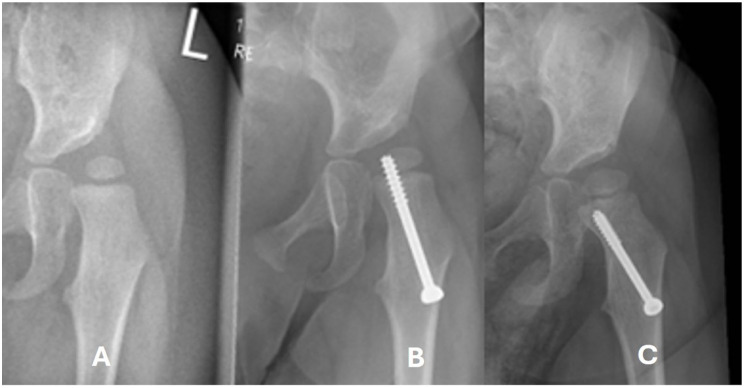
Guided growth around the hip. Radiographs are preoperative (a), direct postoperative (b), and after 14 month follow-up (c). A decrease in coxa valga and hip migration is seen. In (c), continued physeal growth means the screw threads no longer cross the physis, and screw revision is planned.

Most publications on this technique report on its use in children with cerebral palsy. A systematic review^41^ found this technique safe and effective for guiding proximal physeal growth to correct coxa valga deformities, prevent further hip displacement, and reduce hip subluxation.^41,43,44^ It has shown significant improvements in key radiographic measures, including migration percentage (MP), head-shaft angle (HSA), neck-shaft angle, and acetabular index (Figure 4).^32,34,35,41,45^ The HSA correction rates are reported as a mean change of 12.28° (95% CI 11.17–13.39) after 2 years.^41^ Higher correction rates are seen in younger patients and in those with a more eccentric (medial) screw position.^5,35,40^ Overall high success rates are reported, only 5%–21% of children subsequently required pelvic and/or femoral osteotomies after guided growth.^34,35,41^ These studies included children with Gross Motor Function Classification System (GMFCS) levels 3 or higher, a mean MP of at least 33%, and a mean age of 7 years with or without associated soft tissue releases.

While the current literature has not yet defined strict indications, in general, MP below 50%, HSA greater than 145°, and acetabular index (AI) less than 30° are recommended thresholds.^38^ Deformities exceeding these thresholds are unlikely to be completely corrected with guided growth alone, although they may be useful to delay surgery to an older age. Current evidence consists mainly of retrospective single-center cohort studies, and prospective randomized trials are essential to further define the role of this minimally invasive treatment for progressive hip migration in children with CP.^38^

Beyond cerebral palsy, proximal femoral guided growth has been reported in other etiologies. Multiple studies describe its application in children with DDH, addressing associated proximal femur growth disturbances and coxa valga.^31,37,46^ These studies consistently report improvements in proximal femoral morphology with a reduction of the neck valgus.^31,37,46^ Coxa breva often persists, rebound deformity can occur,^36^ and the effect on acetabular development remains uncertain.^36,37^

A small study of children with Hereditary Multiple Exostosis (HME) presenting with coxa valga and hip subluxation demonstrated a positive response to proximal femoral guided growth, comparable to outcomes in children with CP.^32^ This study reported significant decreases in HSA (−12 ± 5°) and MP (−7% ± 8%) over a minimum follow-up of 2 years.^32^

A commonly reported problem during treatment is the physis growing off the screw, which may occur in up to 50% of cases, depending on the duration of treatment and the patient’s age.^15,33,41^ In younger children, due to the small size of the head, it is recommended to place the screw more centrally across the middle quarter of the medial physis. This approach ensures a larger purchase of the screw and reduces the frequency of screw exchange while still successfully tethering the physis.^40^

Another consideration is the potential impact of guided growth on femoral head sphericity. By creating a medial tether in one place and allowing growth on the superolateral side, the sphericity of the femoral head may be altered. A recent study demonstrated changes in the alpha angle suggestive of sphericity loss.^47^ The relevance of femoral head sphericity varies depending on the underlying pathology and the child’s functional status. In children with CP, hip migration often affects non-ambulatory individuals, and sphericity is probably less critical. Alternatively, in ambulatory children with DDH or other hip disorders amendable to guided growth, femoral head sphericity plays a crucial role. In these cases, loss of sphericity might provoke symptoms and early degenerative changes.^48-50^

Further research is required to characterize the effects on sphericity, three-dimensional femoral head shape, function, and patient-reported outcomes. Uncertainty also exists regarding premature (partial) physeal closure and overcorrection after proximal femur guided growth. Careful consideration is warranted when using this technique in ambulatory pediatric patients, as it may have functional implications.

## Knee

The use of guided growth techniques around the knee is frequently indicated for the management of lower limb angular deformities particularly in the coronal plane (genu varum and genu valgum) but also in the sagittal plane to address fixed flexion deformities. The potential for treating rotational deformities is also gaining attention.

### Coronal plane deformities

Angular coronal plane deformities around the knee are encountered relatively frequently in children due to disturbances in skeletal growth. A broad range of idiopathic and non-idiopathic etiologies lead to either varus or valgus deformity, including fractures, Blount’s disease, rickets, infections, and skeletal dysplasia. Depending on the severity, lower-limb angular deformity may lead to cosmetic complaints, functional problems, pain, gait disturbances, and subsequently joint degeneration.^51,52^ Indications for guided growth include persistent or progressive deformities categorized by a mechanical axis in zone 2 or higher according to Stevens, accompanied by pain, patellofemoral instability, and/or gait abnormalities.^53,54^

**Figure 5.**
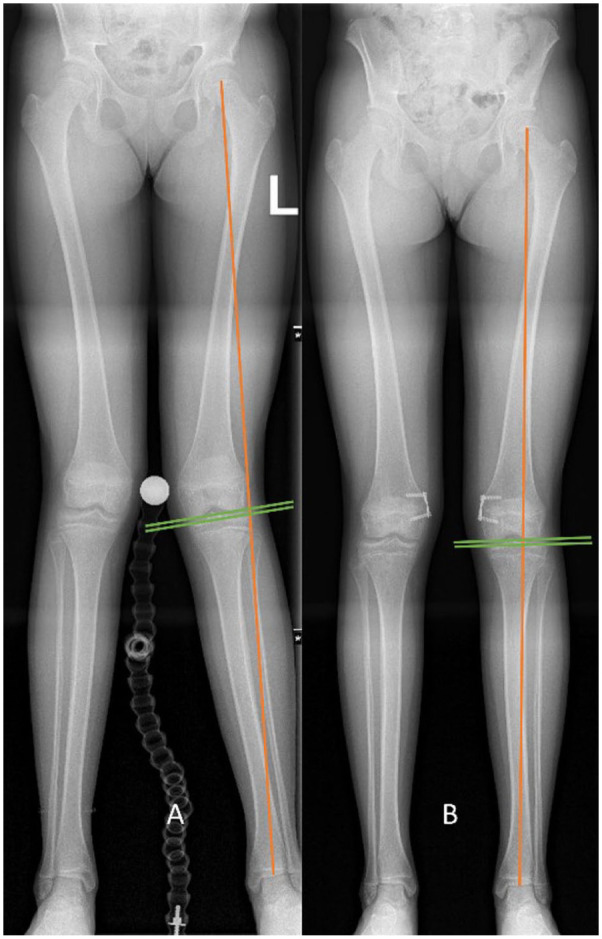
Guided growth of the distal femur to correct the mechanical axis and the genu valgum. Radiographs are preoperative (a) and after 10 month follow-up (b).

Blount staples are a technique with demonstrated efficacy, but the staples are prone to migration. This can be partly mitigated by the use of multiple staples, but the need for revision is common and can be difficult, especially if the staple prongs have also diverged.^12,55,56^ Furthermore, some studies have demonstrated that a single tether is more efficient in correcting angular deformities than multiple tethers.^57^ Therefore, currently, the most widely used guided growth techniques for angular deformities around the knee are tension band plating (Figure 5), flexible staples with a working mechanism similar to TBPs, and PETS.^12,13,58^ TBPs and PETS approaches have been associated with complication rates less than 10%.^7^ PETS have been reported to show faster correction rates compared to TBPs,^59^ and some series report no growth disturbance or physeal bars following screw removal.^60,61^ By contrast, others report ongoing angular correction after screw removal in up to a quarter of patients, suggestive of physeal growth disturbance, especially in those with very eccentrically placed screws.^62^ Overall, both TBPs and PETS are viable temporary hemi-epiphysiodesis techniques. Concerns have been raised regarding the potential development of intra-articular deformities following guided growth. Such changes have been reported in the context of longitudinal correction using dual tension-band plating, particularly at the level of the proximal tibia.^63^ However, using hemiepiphysiodesis for angular correction, these changes appear to be minimal and are unlikely to result in clinically relevant intra-articular deformity.^64^

With relation to screw position in tension band plating, early hypotheses suggested that divergent screws would accelerate correction by immediately engaging the plate and reducing slack.^12,65^ Subsequent biomechanical studies have contradicted this assumption. Parallel screws would result in faster and greater angular correction than divergent screws, likely due to their superior ability to toggle at the plate–screw interface, effectively shifting the center of rotation to a more favorable extraphyseal position.^66,67^ Overall plate size, screw length, and initial screw angle do not appear to significantly impact treatment results.^66–69^ Therefore, it is recommended that surgeons place screws according to anatomic constraints to avoid the joint surface and growth plate, rather than prioritizing screw alignment. Close proximity and convergence of the epiphyseal screw toward the growth plate should probably also be avoided because it bears a higher risk of physeal migration.^70^

The timing of the procedure depends on skeletal age, underlying etiology, and degree of deformity. Sufficient growth should remain to allow for complete deformity correction, and the rate of growth in relation to underlying etiology and age should be considered. Overall correction rates have been reported: 0.87°/month (0.65–1.3) at the distal femur, and 0.72°/month (0.5–1) for proximal tibial deformities.^71,72^ It must be noted, though, that for non-idiopathic deformities with slower growth rates, like skeletal dysplasias, slow correction rates should be anticipated and timing of the procedure adjusted accordingly.^22,72,73^ Furthermore, correction of femoral valgus deformity is reported to be significantly faster than correction of femoral varus deformity. For guided growth of the tibia, no difference in correction rates was found regarding varus versus valgus corrections.^72^

The rebound phenomenon after implant removal around the knee has been widely studied, but it is a phenomenon that is still poorly understood. Definitions vary across the literature; some are based on mechanical axis deviation change (≥3 mm) and others on joint orientation angle change (≥3°–5°) toward the initial deformity after material removal.^74–76^ Despite this variation, most studies report rebound rates up to 50%.^74–78^ Leveille et al.^75^ reported an overall mean change of 6.9° (0–23) in hip-knee-angle (HKA) after implant removal. Rebound was defined as a change of 5°, and those classified as rebound had a mean change of 11.1° (5°–23°) in HKA. Reported risk factors include younger age, valgus deformity, higher deformity correction rate, greater total correction, and underlying pathology.^74–77^ In cases at higher risk of rebound, some overcorrection is probably advantageous; based on the current literature, a specific recommendation on indication and amount of overcorrection cannot be made.

Another option for patients at high risk for rebound deformity is the sleeper plate technique.^79^ This technique involves removing only the metaphyseal screw from the TBP after deformity correction, leaving the plate and epiphyseal screw in place to allow for potential reactivation if rebound occurs. Several studies suggest that the sleeper plate technique can be useful,^79^ although others report substantial risk of ongoing angular correction after metaphyseal screw removal due to tethering—particularly in the tibia, for younger patients, titanium implants, and in patients with HME.^80–82^ Reinsertion of the metaphyseal screw may be technically challenging or impossible due to osseous overgrowth of the plate.^68^ Therefore, the benefits might not outweigh the possible complications associated with this technique, and careful follow-up of alignment is essential.

### Sagittal plane deformities

Fixed flexion deformities around the knee can also be addressed using guided growth techniques. These procedures are commonly performed in patients with neuromuscular conditions but they are not limited to this patient population. A recent systematic review demonstrated a mean angular correction achieved of 14.5° ± 2.2°, representing a 63% decrease in the deformity angle.^83^ On average, the rate of correction was 0.8° ± 0.3°/month, with the desired correction achieved after a mean duration of 19.1 ± 7.7 months. The same review also reported a very low overall complication rate of 7%, with the most reported complication being knee pain that would resolve after physiotherapy and/or hardware removal. Other complications mentioned include infections, hardware loosening, which required revision surgery, and wound dehiscence. No cases of unplanned overcorrection were reported, although rebound was reported in 3% of patients.^83^ The most commonly utilized implants are TBPs and PETS, with a growing preference for PETS due to a lower incidence of implant-related soft tissue irritation.^84^ The optimal surgical timing remains controversial, and although recommendations differ, most suggest that at least 2 years of remaining growth are required to allow correction to occur.^83,85,86^ Others suggest much earlier intervention to facilitate more modulation.

### Rotational deformities

A novel application for guided growth is to correct rotational deformities. Various surgical techniques have been described, all involving implants placed obliquely over the distal femur and/or the proximal tibia physis. It has been reported in animal and small clinical series using TBPs, specifically designed plates, plate–fibertape, and screw-cable constructs.^87–90^

Initial animal studies demonstrate that rotational deformities can be corrected through guided growth.^91–95^ In subsequent clinical studies, an effect averaging between 25° and 30° (±20°–35°) for the femur and 9.5° (±5°–17°) for the tibia is reported over a mean correction period of 12–22 months.^87–89^ It has to be noted that the sample size in these human studies is small, with a total of only 16 patients reported on across the available literature.

Animal studies report a very high risk of rebound, up to approximately 20°, which approaches the amount of initial correction.^96^ Secondary angular deformities and leg length discrepancies have been observed in animal models, with a 4%–7% decrease in longitudinal growth. In the available small sample clinical studies, both rebound and secondary angular deformities have thus far not been observed.^88^ Longitudinal growth discrepancies of approximately 12 mm were estimated for unilateral rotational guided growth.^89^ In studies with bilateral rotational guided growth, no length differences were seen.^88,89^

Overall, based on these reports, rotational guided growth is a promising technique, but warrants further study in correction and rebound rate, optimal implant design, potential secondary deformities, and long-term effects.

## Ankle and foot

### Coronal plane deformities

Guided growth at the distal tibia has been described to correct coronal plane deformities using either medial malleolar transphyseal screws or TBPs placed over the medial side of the distal tibia physis. Ankle valgus is most often seen in posttraumatic cases, neuromuscular disorders, and HME. It can lead to pain, difficulties with bracing, and early ankle osteoarthritis.^97^ Both PETS and TBPs have demonstrated adequate deformity correction in this region. Malleolar screws have been associated with a higher rate of complications: 23% with malleolar screws versus 4% with tension band plating, with screw migration being most common.^98^ While some authors have suggested that the correction rate is faster with screws, the evidence is conflicting.^53,98,99^ Chang et al.^100^ found a correction rate of 0.37° ± 0.04°/month for transphyseal screws. Stevens et al.^99^ found a correction rate of 0.60°/month (range 0.15°–1.6°/month) for tension band plating. However, Driscoll et al.^98^ compared both treatments and found that the mean correction rate was faster in the malleolar screw-treated ankles (0.55° ± 0.41°/month) than in the TBP-treated ankles (0.36° ± 0.32°/month).

Regarding timing and implant size, skeletal age must be considered since patients who are too young may not have a sufficiently developed distal tibial epiphysis to facilitate guided growth implants.^101^ Medial malleolar transphyseal screws are feasible from age 4, while TBPs are feasible from age 6.^98,99^ Another factor to take into account is the rebound of the deformity. For ankle valgus, it has been reported that rebound occurs in 80% of ankles with an average rebound rate of 0.28° ± 0.08°/month.^100^ Therefore, timing should be determined by skeletal age, size of the distal tibial epiphysis, the desired speed and rate of correction, and underlying disease.

**Figure 6.**
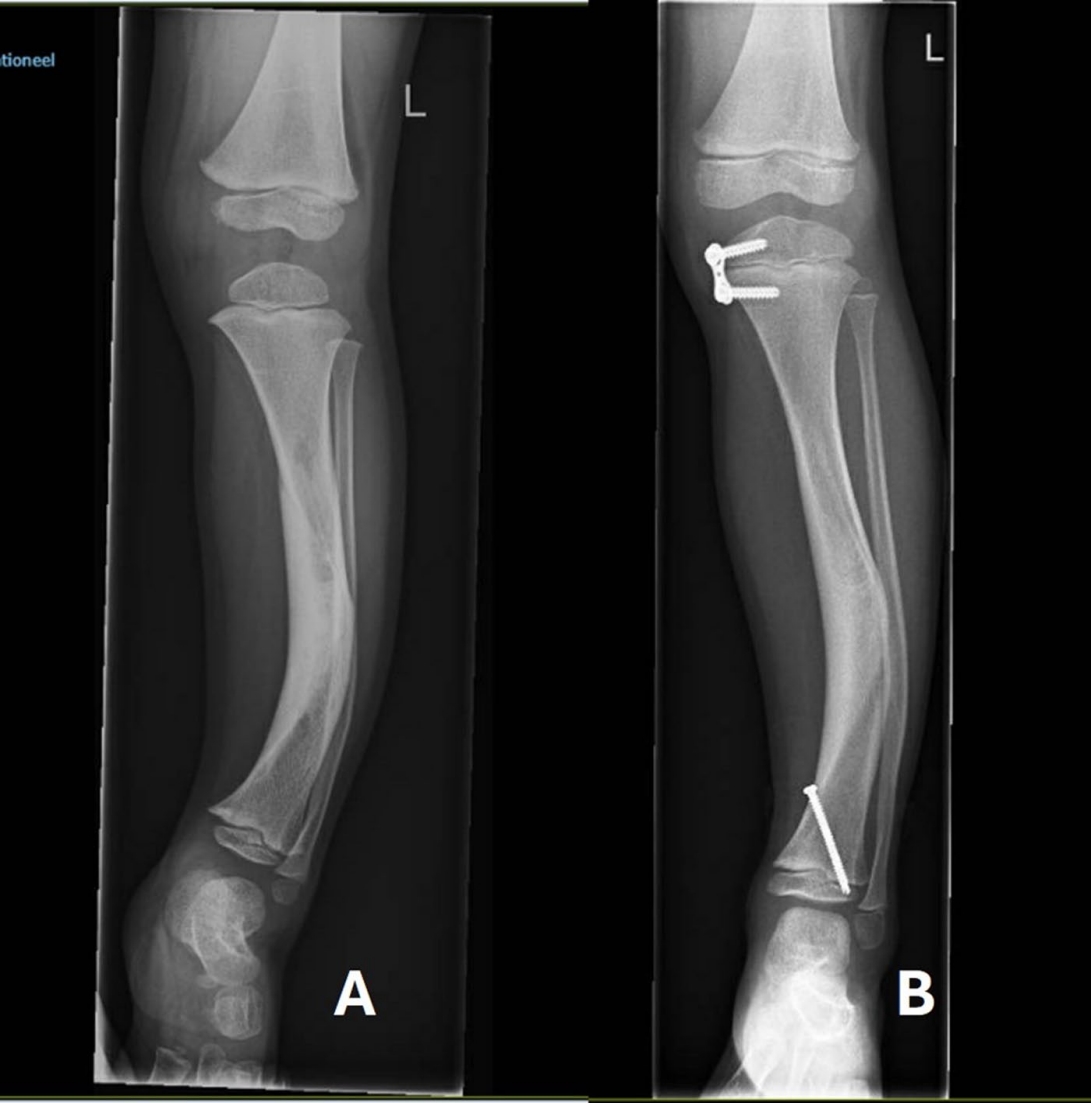
Radiographs of anterolateral bowing of the tibia in a patient with neurofibromatosis 1. Preoperative (a) and after 2-year follow-up (b). Distally, the choice of implant used was based on anatomical landmarks; the tension band plate was not feasible on the lateral side of the tibia due to the presence of the fibula.

A novel indication for guided growth in the lower leg is anterolateral bowing of the tibia (Figure 6), a deformity associated with the development of congenital pseudarthrosis of the tibia (CPT). In two series comprising a total of 13 patients with anterolateral bowing, it has been reported that improvement of alignment using TBPs at the distal tibia could reduce the risk of developing a (re-)fracture and pseudarthrosis.^102,103^ Only 1 of the 13 reported patients experienced a refracture, and none of the pre-fracture patients experienced a tibial fracture during the 5-year follow-up period.^102,103^ Although early results are promising, larger series and longer follow-up are needed to further determine the success rate and identify specific pre-fracture CPT types that are suitable for this minimally invasive technique.

### Sagittal plane deformities

**Figure 7.**
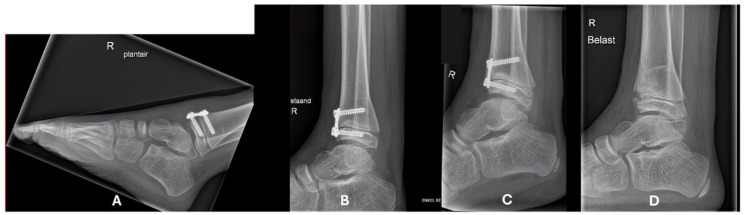
Guided growth for equinus deformities. Radiographs are directly postoperative (a), after 14 months of follow-up in standing position (b), after 23 months of follow-up in standing position (c), and at 40 months of follow-up after removal of TBP in standing position (d). A gradual increase in the anterior distal tibial angle can be observed, with a corresponding clinical improvement in ankle dorsiflexion.

Guided growth may also be a viable option for managing equinus deformities (Figure 7). This is mostly reported in patients treated via the Ponseti method for clubfoot. Anterior distal tibial hemiepiphysiodesis using TBPs has demonstrated safety and efficacy in treating recurrent equinus deformity with or without associated flat top talus deformity.^104^ The correction rates and changes in anterior distal tibial angle (ADTA) have been reported in various studies.^104–106^ Overall, a mean correction rate of 0.6°–1.2°/month was observed, with a mean reduction in ADTA of 11.7°–17.3°.^104–106^

Rebound is a common phenomenon in surgically treated clubfeet with guided growth. Besselaar^107^ showed in 32 clubfoot patients (46 feet) that despite good initial correction at a median follow-up of 20 months after plate removal, both ADTA and ankle dorsiflexion showed significant rebound towards preoperative values. When using this approach, repeat interventions and/or timing towards the end of growth should be considered.

### Angular deformities of the hallux

The management of juvenile hallux valgus (JHV) remains a topic of debate due to the limited evidence base supporting specific interventions. Notably, there is a lack of studies directly comparing surgical and non-surgical approaches. Guided growth at the first metatarsal for JHV is an option and involves methods such as stapling, screws, or hemi-physeal removal using drills and curettes.^5,108^ A systematic review reported on 147 feet and demonstrated modest radiological improvements with a mean hallux valgus angle decreasing from 29.2° ± 3.7° to 23.8° ± 4.5°, and intermetatarsal angle improving from 13.9° ± 1.1° to 11.4° ± 1.2°. Overall, 14.2% experienced complications, including recurrence and the need for revision surgery.^108^ Because of the relatively slow correction rates, the procedure may be effective in mild to moderate deformities when performed early, as younger patients demonstrated better outcomes. Current recommendations suggest performing guided growth with at least 2 years of growth remaining to maximize corrective potential.^5,108^

### Cavovarus foot deformity

In a subgroup of patients with cavovarus deformity, first metatarsal correction with or without additional soft tissue procedures can be a good option. It has been shown that in patients with sufficient remaining growth, dorsal hemiepiphysiodesis of the first metatarsal can be a less invasive alternative to osteotomy of the base of the first metatarsal. In a pilot study of 13 children treated with dorsal hemiepiphysiodesis plus plantar fascia release, clinical improvement occurred at 28 months. Hindfoot alignment improved from 6° varus to 5° valgus.^109^ Similarly, in 8 children undergoing dorsal hemiepiphysiodesis with plantar fascia release, significant radiographic correction was achieved at a median 4.3-year follow-up. Moreau–Costa–Bartani angle: increased from 112° to 120°, whereas the Meary angle decreased from 10° to 5°, with no complications described.^110^ Optimal timing is around ages 8–11 years, when hindfoot varus remains flexible and first metatarsal physeal growth persists. These early results are promising, but larger series and longer follow-up are necessary to determine predictors of success.

## Guided growth in the upper extremity

Although guided growth is well established for lower extremity applications, its use in the upper extremity is less widespread in current clinical practice.^111^ Nevertheless, a limited but growing body of evidence supports its potential use in addressing upper limb deformities. Application to the distal humerus, distal radius, and ulna has been described.

## Humerus

Distal humerus deformity leading to cubitus valgus or varus can be either congenital or posttraumatic, with the latter being the most common. Depending on the type and severity of deformity, they can lead to significant functional and cosmetic issues. Due to the limited growth potential of the distal humerus, such deformities are rarely corrected by normal growth and often require osteotomy. Guided growth can be a less invasive option, although the evidence remains limited.

**Figure 8.**
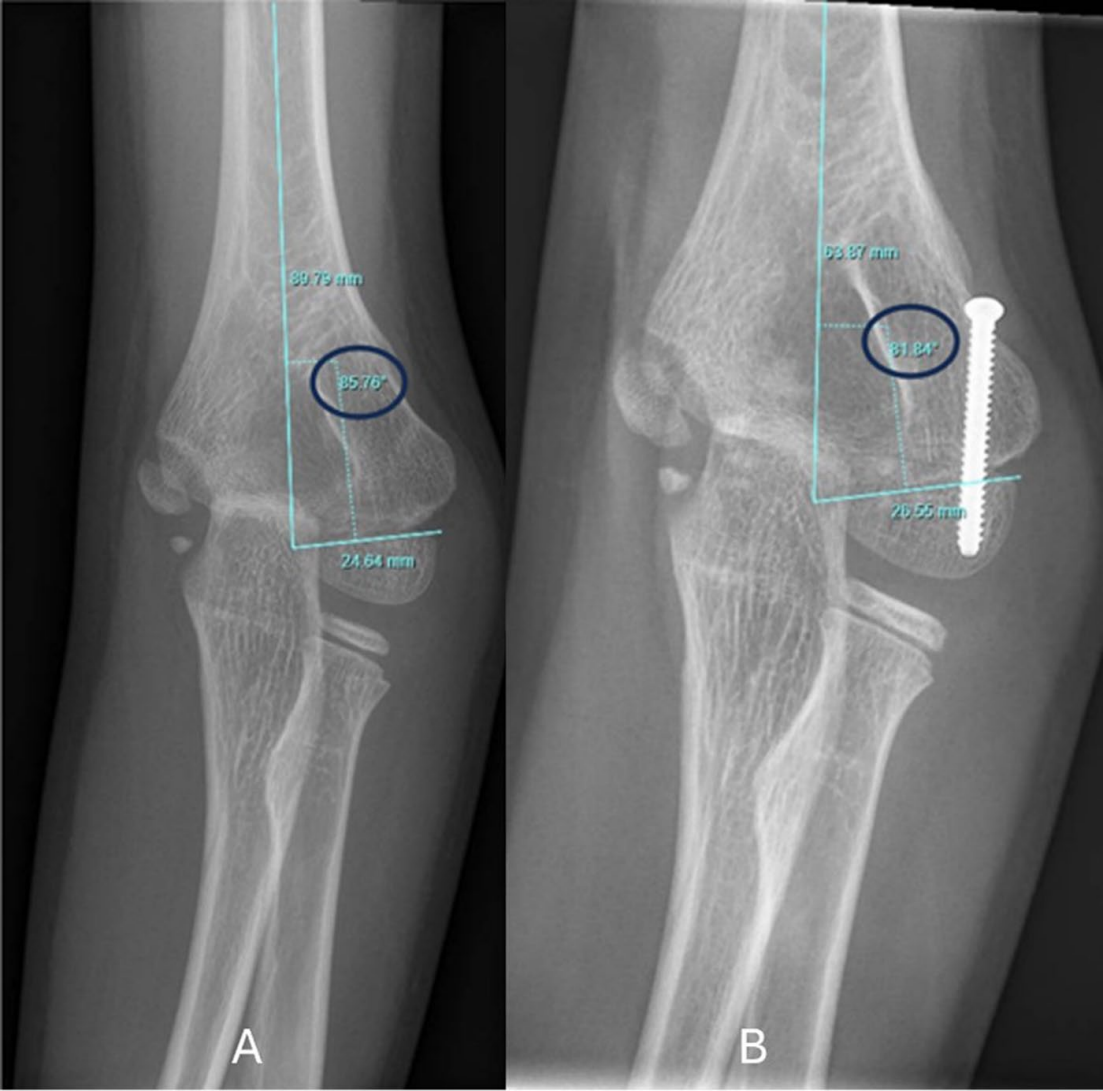
Guided growth around the elbow. Radiographs preoperative (a) and after 1-year follow-up (b). B shows the Baumann angle has improved (circle).

Two guided growth techniques that have been explored in small experimental studies are PETS (Figure 8) and tension band plating.^112,113^ While the number of cases studied is limited and results vary, some results show moderately positive outcomes.^113–115^

Soldado et al. investigated the use of medial oblique PETS in five children with cubitus varus. No correction of deformity was observed in this study. This lack of improvement may have been due to the short duration of follow-up.^112^ In addition, two case reports have described improvements in the carrying angle following treatment with tension band plating. Both reports show some radiological and clinical improvement in cubitus varus deformity, but the reported improvements may fall within the limits of measurement error.^114,115^

Martínez-Álvarez et al. published a series of lateral distal humeral hemiepiphysiodesis using TBPs for posttraumatic cubitus varus correction in 15 patients. Significant improvements were found in the radiological measurements humero-ulnar angle (HUA), Baumann angle (BA), and shaft-condylar angle (SCA), as well as in the clinical measurements of the carrying angle. Correction rates per year varied from 2.4° for HUA, 1.5° for BA, and 1.4° for SCA. They could not find any correlation between age at intervention and the degree of correction (for radiographic and clinical parameters), but they did find a correlation between longer implant duration and clinical correction rates. Thirty-three percent of patients required additional surgery due to aseptic screw loosening, highlighting the need for careful surgical technique and close follow-up.^113^

Some correction of guided growth at the distal humerus might be expected, but due to the slow growth speed in this region, correction is expected to be slow. Its use is limited to mild-to-moderate deformities and to younger children with sufficient growth remaining. Based on current evidence, no clear guidelines can be formulated.

## Radius

The distal radius physis contributes the majority of longitudinal growth of the radius, and disruptions to this growth plate can result in deformities such as positive ulnar variance and distal radioulnar joint instability. Common causes of such disturbances include multiple hereditary exostoses, Madelung deformity, and physeal fractures. The relatively high growth rates make deformities in this region good candidates for guided growth, but the anatomical features necessitate adaptation to smaller implants like extraperiostal staples and small customized plates.^116,117^

Two studies describe the results of guided growth of the distal radius in patients with increased radial inclination and secondary ulnar shift of the carpus, in series with predominantly HME patients. Both measured changes in radial articular angle (RAA), carpal slip (CS), and ulnar tilt (UT) and identified significant improvements. Kelly and James^117^ unfortunately did not report follow-up time and correction rates, but incomplete correction was frequent, and the authors hypothesized that the procedure might have been undertaken too late. Soler-Jimenez et al. also demonstrated significant improvements in RAA (0.6°/month), CS (1.2°/month), and UT (0.6°/month), at an average follow-up of 28.7 ± 8.9 months. At the time of implant removal, most patients still had open physes, and removal was performed either following intentional overcorrection or upon reaching skeletal maturity. Reported complications included deformity rebound in two cases, neither of which required additional surgical intervention, and one case of screw breakage that did require reoperation.^116^

## Ulna

Guided growth has been reported to be useful for positive ulnar variance. Scheider et al. demonstrated the utility of performing temporary epiphysiodesis using an intraoperatively customized small plate over the distal ulna physis. They report a reduction in ulnar variance from +3.9 mm (+1.9 to +6.1) to +0.1 mm (–3.2 to +5.0) and reduced ulnocarpal wrist complaints after an average correction time of 2.3 years in a cohort of seven wrists.^118^ The authors concluded that this technique is effective in correcting positive ulnar variance without causing irreversible physeal damage.

Madelung deformity, characterized by an abnormality of the volar-ulnar portion of the distal radial physis,^119^ may also benefit from guided growth techniques. In a study by Farr et al.,^120^ performed on children with Madelung deformity, 10 wrists out of 41 received an ulnar epiphysiodesis. Of these 10 wrists, none required a second intervention for deformity correction. The authors postulated that ulnar epiphysiodesis may be considered in skeletally immature children older than 10 years of age with Madelung deformity, but correction rates or influencing factors were not provided.

## Conclusion

Guided growth represents an essential tool in the treatment of pediatric orthopedic conditions, offering a minimally invasive approach to correct limb deformities. This technique leverages the child’s own growth potential to gradually restore normal alignment, minimizing the need for more invasive surgical procedures.

Most evidence is available for coronal plane deformities around the knee. For this indication, guided growth outcomes are generally favorable. Clear predictors for correction rate and treatment success are available to guide treatment decisions, although rebound after implant removal remains difficult to predict.

Several new indications have emerged throughout the growing skeleton. Guided growth of the proximal femur to treat pediatric hip disorders is gaining popularity, especially in the treatment of neuromuscular hip migration. Promising early results have been reported; however, further validation, including randomized controlled trials, studies assessing patient‑reported outcomes, and changes in femoral head sphericity, is required. Rotational guided growth is another emerging technique, aiming for minimal invasive rotational deformity correction. However, supporting evidence from clinical studies to date is limited. Future rotational guided growth research should focus on correction and rebound rate, optimal implant design, potential secondary deformities, and long-term effects. Also, guided growth for upper extremity deformity is currently based on relatively limited evidence and requires further investigation on optimal patient selection, surgical techniques, and expected outcomes.

In all guided growth procedures, timing is essential to optimize outcomes and should be individually tailored. Considerations should include the underlying etiology, severity of deformity, growth rate of the treated physis, and skeletal age, ensuring sufficient growth potential to allow for complete deformity correction. While challenges remain, ongoing research and technological advancements continue to expand applications and improve outcomes of guided growth in children.

## Supplemental Material

sj-pdf-1-cho-10.1177_18632521261456090 – Supplemental material for Guided growth: Current concepts and novel techniques for harnessing a child’s growth potential Supplemental material, sj-pdf-1-cho-10.1177_18632521261456090 for Guided growth: Current concepts and novel techniques for harnessing a child’s growth potential by Merel C. R. Roelen, Christiaan J. A. van Bergen, Mark F. Siemensma, Deborah M. Eastwood, Ignacio Sanpera and Jaap J. Tolk in Journal of Children's Orthopaedics

## References

[1] Bartonicek J Nanka O. The true history of the Hueter-Volkmann law. Int Orthop 2024; 48: 2755–2762. 39083236 10.1007/s00264-024-06254-w PMC11422464

[2] Hert J. Regulace Rustu Dlouh’ych Kost’i Do D’elky [Regulation of the longitudinal growth of long bones] Acta Chir Orthop Traumatol Cech 1964; 31: 85–91. 14147593

[3] Hueter C. Anatomische Studien an den Extremitätengelenken Neugeborener und Erwachsener. Archiv Pathol Anat (Virchows Archiv) 1863; 26: 484–519.

[4] Volkmann R. Die Krankheiten der Bewegungsorgane. In: von Pitha FR Billroth T (eds) Handbuch der allgemeinen und speziellen Chirurgie. Stuttgart: Ferdinand Enke, 1865, pp.350–351.

[5] Cappello T. Expanded indications for guided growth in pediatric extremities. J Pediatr Orthop Soc North Am 2021; 3: 217.

[6] Gottliebsen M Shiguetomi-Medina JM Rahbek O Moller-Madsen B. Guided growth: mechanism and reversibility of modulation. J Child Orthop 2016; 10: 471–477. 27826908 10.1007/s11832-016-0778-9 PMC5145828

[7] Yang I Gottliebsen M Martinkevich P , et al. Guided growth: current perspectives and future challenges. JBJS Rev 2017; 5: e1. 10.2106/JBJS.RVW.16.00115 29112518

[8] Phemister DB. Operative arrestment of longitudinal growth of bones in the treatment of deformities. J Bone Joint Surg 1933; 15: 1–15.

[9] Hubbard EW Cherkashin A Samchukov M , et al. The evolution of guided growth for lower extremity angular correction. J Pediatr Orthop Soc North Am 2023; 5: 738. 10.55275/JPOSNA-2023-738 PMC12088150 40433335

[10] Haas SL. Retardation of bone growth by a wire loop. JBJS 1945; 27: 25–36.

[11] Blount WP Clarke GR. Control of bone growth by epiphyseal stapling; a preliminary report. J Bone Joint Surg Am 1949; 31A: 464–478. 18153890

[12] Stevens PM. Guided growth for angular correction: a preliminary series using a tension band plate. J Pediatr Orthop 2007; 27: 253–259. 17414005 10.1097/BPO.0b013e31803433a1

[13] Metaizeau JP Wong-Chung J Bertrand H Pasquier P. Percutaneous epiphysiodesis using transphyseal screws (PETS). J Pediatr Orthop 1998; 18: 363–369. 9600565

[14] Lascombes P Omeroglu H. Long bone deformity correction and bone lengthening procedures. J Child Orthop 2016; 10: 469–470. 27933570 10.1007/s11832-016-0796-7 PMC5145846

[15] Hsu PJ Lee CC Lin SC , et al. Guided growth versus varus osteotomy for type II avascular necrosis following surgery for developmental dysplasia of the hip. Bone Joint J 2022; 104-B: 902–908. 35775168 10.1302/0301-620X.104B7.BJJ-2021-1308.R1

[16] Stevens PM. Guided growth for deformity correction. Oper Tech Orthop 2011; 21: 197–202.

[17] Eastwood DM Sanghrajka AP. Guided growth: recent advances in a deep-rooted concept. J Bone Joint Surg Br 2011; 93: 12–18. 21196537 10.1302/0301-620X.93B1.25181

[18] Hucke L Holder J van Drongelen S , et al. Influence of tension-band plates on the mechanical loading of the femoral growth plate during guided growth due to coronal plane deformities. Front Bioeng Biotech 2023; 11: 1165963. 10.3389/fbioe.2023.1165963 PMC10321528 37415789

[19] Inan M Senaran H Domzalski M , et al. Unilateral versus bilateral peri-ilial pelvic osteotomies combined with proximal femoral osteotomies in children with cerebral palsy: perioperative complications. J Pediatr Orthop 2006; 26: 547–550. 16791078 10.1097/01.bpo.0000226277.08825.c2

[20] McNerney NP Mubarak SJ Wenger DR. One-stage correction of the dysplastic hip in cerebral palsy with the San Diego acetabuloplasty: results and complications in 104 hips. J Pediatr Orthop 2000; 20: 93–103. 10641697

[21] Horn A Wright J Bockenhauer D , et al. The orthopaedic management of lower limb deformity in hypophosphataemic rickets. J Child Orthop 2017; 11: 298–305. 28904636 10.1302/1863-2548.11.170003 PMC5584499

[22] Tolk JJ Kessling LM Yeo A , et al. Guided growth for the correction of angular lower limb deformity: correction rates for idiopathic and non-idiopathic aetiologies. J Child Orthop 2026; 20: 159–166. 41696355 10.1177/18632521251411732 PMC12904809

[23] Villemure I Stokes IA. Growth plate mechanics and mechanobiology. A survey of present understanding. J Biomech 2009; 42: 1793–1803. 19540500 10.1016/j.jbiomech.2009.05.021 PMC2739053

[24] D’Andrea CR Alfraihat A Singh A , et al. Part 1. Review and meta-analysis of studies on modulation of longitudinal bone growth and growth plate activity: a macro-scale perspective. J Orthop Res 2021; 39: 907–918. 33377536 10.1002/jor.24976

[25] Kaviani R Londono I Parent S , et al. Compressive mechanical modulation alters the viability of growth plate chondrocytes in vitro. J Orthop Res 2015; 33: 1587–1593. 26019113 10.1002/jor.22951

[26] D’Andrea CR Alfraihat A Singh A , et al. Part 2. Review and meta-analysis of studies on modulation of longitudinal bone growth and growth plate activity: a micro-scale perspective. J Orthop Res 2021; 39: 919–928. 33458882 10.1002/jor.24992

[27] Frost HM. A chondral modeling theory. Calcif Tissue Int 1979; 28: 181–200. 92358 10.1007/BF02441236

[28] Hamrick MW. A chondral modeling theory revisited. J Theor Biol 1999; 201: 201–208. 10600363 10.1006/jtbi.1999.1025

[29] Akyuz E Braun JT Brown NA , et al. Static versus dynamic loading in the mechanical modulation of vertebral growth. Spine (Phila Pa 1976) 2006; 31: E952–E958. 10.1097/01.brs.0000248810.77151.22 17139211

[30] Mente PL Aronsson DD Stokes IA Iatridis JC. Mechanical modulation of growth for the correction of vertebral wedge deformities. J Orthop Res 1999; 17: 518–524. 10459757 10.1002/jor.1100170409

[31] Torode IP Young JL. Caput valgum associated with developmental dysplasia of the hip: management by transphyseal screw fixation. J Child Orthop 2015; 9: 371–379. 26362171 10.1007/s11832-015-0681-9 PMC4619369

[32] Hung TY Wu KW Lee CC , et al. Guided growth improves coxa valga and hip subluxation in children with hereditary multiple exostoses. J Pediatr Orthop 2023; 43: e67–e73. 10.1097/BPO.0000000000002296 36509457

[33] Lee WC Kao HK Yang WE , et al. Guided growth of the proximal femur for hip displacement in children with cerebral palsy. J Pediatr Orthop 2016; 36: 511–515. 25887815 10.1097/BPO.0000000000000480

[34] Hsieh HC Wang TM Kuo KN , et al. Guided growth improves coxa valga and hip subluxation in children with cerebral palsy. Clin Orthop Relat Res 2019; 477: 2568–2576. 31425278 10.1097/CORR.0000000000000903 PMC6903837

[35] Portinaro N Turati M Cometto M , et al. Guided growth of the proximal femur for the management of hip dysplasia in children with cerebral palsy. J Pediatr Orthop 2019; 39: e622–e628. 10.1097/BPO.0000000000001069 31393306

[36] Peng SH Lee WC Kao HK , et al. Guided growth for caput valgum in developmental dysplasia of the hip. J Pediatr Orthop B 2018; 27: 485–490. 29851711 10.1097/BPB.0000000000000529

[37] Shin CH Hong WK Lee DJ , et al. Percutaneous medial hemi-epiphysiodesis using a transphyseal screw for caput valgum associated with developmental dysplasia of the hip. BMC Musculoskelet Disord 2017; 18: 451. 29137619 10.1186/s12891-017-1833-5 PMC5686794

[38] van Stralen RA Roelen MCR Moerman S , et al. GUIDANCE study: guided growth of the proximal femur to prevent further hip migration in patients with cerebral palsy-study protocol for a multicentre randomised controlled trial. BMJ Open 2024; 14: e091073. 10.1136/bmjopen-2024-091073 PMC11647319 39663160

[39] Davids J. Proximal femur guided growth for the management of hip dysplasia in children with cerebral palsy. J Pediatr Orthop Soc North Am 2021; 3: 245. 10.1097/BPO.0000000000001069 31393306

[40] Hsu PJ Wu KW Lee CC , et al. Does screw position matter for guided growth in cerebral palsy hips? Bone Joint J 2020; 102-B: 1242–1247. 32862682 10.1302/0301-620X.102B9.BJJ-2020-0340.R1

[41] Lebe M van Stralen RA Buddhdev P. Guided growth of the proximal femur for the management of the ‘hip at risk’ in children with cerebral palsy-a systematic review. Children (Basel) 2022; 9: 609. 35626786 10.3390/children9050609 PMC9140189

[42] Zakrzewski AM Carl JR McCarthy JJ. Proximal femoral screw hemiepiphysiodesis in children with cerebral palsy improves the radiographic measures of hip subluxation. J Pediatr Orthop 2022; 42: e583–e589. 10.1097/BPO.0000000000002152 35452015

[43] Chang CH Chi CH Lee ZL. Progressive coxa vara by eccentric growth tethering in immature pigs. J Pediatr Orthop B 2006; 15: 302–306. 16751743 10.1097/01202412-200607000-00014

[44] McCarthy JJ Noonan KJ Nemke B , et al. Guided growth of the proximal femur: a pilot study in the lamb model. J Pediatr Orthop 2010; 30: 690–694. 20864854 10.1097/BPO.0b013e3181edef71

[45] Galan-Olleros M Munoz de la Espada M Garcia-Fernandez J , et al. Unilateral hip reconstruction combined with contralateral guided growth versus bilateral reconstruction in children with cerebral palsy and unilateral hip displacement. J Pediatr Orthop B 2025; 35: 178–185. 41362092 10.1097/BPB.0000000000001310

[46] Agus H Onvural B Kazimoglu C , et al. Medial percutaneous hemi-epiphysiodesis improves the valgus tilt of the femoral head in developmental dysplasia of the hip (DDH) type-II avascular necrosis. Acta Orthop 2015; 86: 506–510. 25907982 10.3109/17453674.2015.1037222 PMC4513608

[47] Chiu KC Lee CC Wu KW , et al. Outcome and femoral head deformity following hip guided growth in children with cerebral palsy at skeletal maturity. J Pediatr Orthop 2025; 45: 423–430. 40178803 10.1097/BPO.0000000000002964 PMC12233175

[48] Stulberg SD Cooperman DR Wallensten R. The natural history of Legg-Calve-Perthes disease. J Bone Joint Surg Am 1981; 63: 1095–1108. 7276045

[49] Thomas GE Palmer AJ Batra RN , et al. Subclinical deformities of the hip are significant predictors of radiographic osteoarthritis and joint replacement in women. A 20 year longitudinal cohort study. Osteoarthritis Cartilage 2014; 22: 1504–1510. 25047637 10.1016/j.joca.2014.06.038

[50] Casartelli NC Maffiuletti NA Valenzuela PL , et al. Is hip morphology a risk factor for developing hip osteoarthritis? A systematic review with meta-analysis. Osteoarthritis Cartilage 2021; 29: 1252–1264. 34171473 10.1016/j.joca.2021.06.007

[51] Brouwer GM van Tol AW Bergink AP , et al. Association between valgus and varus alignment and the development and progression of radiographic osteoarthritis of the knee. Arthritis Rheum 2007; 56: 1204–1211. 17393449 10.1002/art.22515

[52] Wu KW Lee WC Ho YT , et al. Balance control and lower limb joint work in children with bilateral genu valgum during level walking. Gait Posture 2021; 90: 313–319. 34564004 10.1016/j.gaitpost.2021.09.187

[53] Masquijo JJ Artigas C de Pablos J. Growth modulation with tension-band plates for the correction of paediatric lower limb angular deformity: current concepts and indications for a rational use. EFORT Open Rev 2021; 6: 658–668. 34532073 10.1302/2058-5241.6.200098 PMC8419796

[54] Stevens PM MacWilliams B Mohr RA. Gait analysis of stapling for genu valgum. J Pediatr Orthop 2004; 24: 70–74. 14676537 10.1097/00004694-200401000-00013

[55] Kumar A Gaba S Sud A , et al. Comparative study between staples and eight plate in the management of coronal plane deformities of the knee in skeletally immature children. J Child Orthop 2016; 10: 429–437. 27417295 10.1007/s11832-016-0758-0 PMC5033777

[56] Hosseinzadeh P Ross DR Walker JL , et al. Three methods of guided growth for pediatric lower extremity angular deformity correction. Iowa Orthop J 2016; 36: 123–127. 27528848 PMC4910790

[57] Sanpera I Jr Raluy-Collado D Frontera-Juan G , et al. Guided growth: the importance of a single tether. An experimental study. J Pediatr Orthop 2012; 32: 815–820. 23147625 10.1097/BPO.0b013e31824b755a

[58] Koob S Kehrer M Hettchen M , et al. Temporary epiphysiodesis using the FlexTack implant (tension band) featuring a modified explantation technique. Temporare Epiphyseodese mit dem FlexTack-Implantat (“Tension Band”) unter Anwendung eines modifizierten Explantationsverfahrens. Oper Orthop Traumatol 2018; 30: 359–368. 29907912 10.1007/s00064-018-0553-9

[59] Sabry AO Genedy MKA Abouelwafa S , et al. Percutaneous epiphysiodesis transphyseal screw versus tension-band plating as hemiepiphysiodesis in treating coronal angular knee deformities: a systematic review and meta-analysis of comparative studies. BMC Musculoskelet Disord 2025; 26: 355. 40217231 10.1186/s12891-025-08540-z PMC11987184

[60] Neal KM Kiebzak GM. Epiphyseal-entry cannulated screws for temporary guided growth of the knees: a retrospective review of 89 cases. J Pediatr Orthop B 2024; 33: 114–118. 37610093 10.1097/BPB.0000000000001118

[61] Abdelaziz TH Ghaly N Fayyad TA , et al. Transphyseal hemiepiphysiodesis: is it truly reversible? J Pediatr Orthop 2024; 44: 619–625. 39187967 10.1097/BPO.0000000000002790

[62] Shim JS Ko KR Lim KS Na S. Factors affecting postoperative courses after removal of transphyseal screws inserted for correction of genu valgum. J Pediatr Orthop 2024; 44: e411–e418. 10.1097/BPO.0000000000002660 38477319

[63] Tolk JJ Merchant R Calder PR , et al. Tension-band plating for leg-length discrepancy correction. Strateg Trauma Limb 2022; 17: 19–25. 10.5005/jp-journals-10080-1547 PMC9166256 35734032

[64] Hvidberg E Antfang C Gosheger G , et al. Morphology of the knee after guided growth using tension-band devices: a retrospective multicenter study of 222 limbs and 285 implants. Acta Orthop 2023; 94: 609–615. 38153250 10.2340/17453674.2023.34902 PMC10755675

[65] Burghardt RD Herzenberg JE Standard SC , et al. Temporary hemiepiphyseal arrest using a screw and plate device to treat knee and ankle deformities in children: a preliminary report. J Child Orthop 2008; 2: 187–197. 19308576 10.1007/s11832-008-0096-y PMC2656802

[66] Schoenleber SJ Iobst CA Baitner A , et al. The biomechanics of guided growth: does screw size, plate size, or screw configuration matter? J Pediatr Orthop B 2014; 23: 122–125. 24322536 10.1097/BPB.0000000000000026

[67] Eltayeby HH Iobst CA Herzenberg JE. Hemiepiphysiodesis using tension band plates: does the initial screw angle influence the rate of correction? J Child Orthop 2019; 13: 62–66. 30838077 10.1302/1863-2548.13.180086 PMC6376435

[68] Raluy-Collado D Sanpera IJ Frontera-Juan G , et al. Screw length in the guided growth method. Arch Orthop Trauma Surg 2012; 132: 1711–1715. 22990385 10.1007/s00402-012-1615-3

[69] Kim NT Kwon SS Choi KJ , et al. Effect of Screw Configuration on the Rate of Correction for Guided Growth Using the Tension-band Plate. J Pediatr Orthop 2021; 41: e899–e903. 10.1097/BPO.0000000000001970 34534159

[70] Galan-Olleros M Sanchez Del Saz J Miranda-Gorozarri C , et al. Physeal migration during knee-guided growth with tension band plates: influence of implant position. J Pediatr Orthop 2024; 44: e174–e183. 10.1097/BPO.0000000000002583 38047324

[71] Kumar S Sonanis SV. Growth modulation for coronal deformity correction by using Eight Plates-Systematic review. J Orthop 2018; 15: 168–172. 29657461 10.1016/j.jor.2018.01.022 PMC5895900

[72] Danino B Rodl R Herzenberg JE , et al. Growth modulation in idiopathic angular knee deformities: is it predictable? J Child Orthop 2019; 13: 318–323. 31312272 10.1302/1863-2548.13.190033 PMC6598046

[73] Danino B Rodl R Herzenberg JE , et al. Guided growth: preliminary results of a multinational study of 967 physes in 537 patients. J Child Orthop 2018; 12: 91–96. 29456760 10.1302/1863-2548.12.170050 PMC5813131

[74] Farr S Alrabai HM Meizer E , et al. Rebound of frontal plane malalignment after tension band plating. J Pediatr Orthop 2018; 38: 365–369. 27574955 10.1097/BPO.0000000000000846

[75] Leveille LA Razi O Johnston CE. Rebound deformity after growth modulation in patients with coronal plane angular deformities about the knee: who gets it and how much? J Pediatr Orthop 2019; 39: 353–358. 31305378 10.1097/BPO.0000000000000935

[76] Ramazanov R Ozdemir E Yilmaz G , et al. Rebound phenomenon after hemiepiphysiodesis: determination of risk factors after tension band plate removal in coronal plane deformities of lower extremities. J Pediatr Orthop B 2021; 30: 52–58. 32732797 10.1097/BPB.0000000000000786

[77] Park SS Kang S Kim JY. Prediction of rebound phenomenon after removal of hemiepiphyseal staples in patients with idiopathic genu valgum deformity. Bone Joint J 2016; 98-B: 1270–1275. 27587531 10.1302/0301-620X.98B9.37260

[78] Haider Z Shah N Acquaah F , et al. Guided growth for genu valgum in mucopolysaccharidoses: beware the rebound. J Pediatr Orthop. Epub ahead of print 15 July 2025. DOI: 10.1097/BPO.0000000000003048. 40660484

[79] Keshet D Katzman A Zaidman M , et al. Removal of metaphyseal screw only after hemiepiphysiodesis correction of coronal plane deformities around the knee joint: is this a safe and advisable strategy? J Pediatr Orthop 2019; 39: e236–e239. 10.1097/BPO.0000000000001257 30222639

[80] Gerges M Messner J Lim B , et al. Efficacy and safety of “sleeper plate” in temporary hemiepiphysiodesis and the observation of “tethering”. J Pediatr Orthop 2022; 42: e762–e766. 10.1097/BPO.0000000000002184 35605208

[81] Bakircioglu S Kolac UC Yigit YA , et al. Does the sleeper plate application for temporary epiphysiodesis make life easier or complicated? Increased risk of tethering. J Pediatr Orthop 2023; 43: 572–577. 37526124 10.1097/BPO.0000000000002489

[82] Retzky J Pascual-Leone N Cirrincione P , et al. The perils of sleeper plates in multiple hereditary exostosis: tibial deformity overcorrection due to tether at empty metaphyseal hole. J Pediatr Orthop 2023; 43: 471–474. 37469302 10.1097/BPO.0000000000002458 PMC10402878

[83] Al Badi H Lorange JP Alzeedi M , et al. Distal femur anterior hemiepiphysiodesis for fixed knee flexion deformity in neuromuscular patients: a systematic review. JBJS Rev 2023; 11: 00001. 10.2106/JBJS.RVW.23.00001 37276266

[84] Stiel N Babin K Vettorazzi E , et al. Anterior distal femoral hemiepiphysiodesis can reduce fixed flexion deformity of the knee: a retrospective study of 83 knees. Acta Orthop 2018; 89: 555–559. 29902104 10.1080/17453674.2018.1485418 PMC6202731

[85] Palocaren T Thabet AM Rogers K , et al. Anterior distal femoral stapling for correcting knee flexion contracture in children with arthrogryposis–preliminary results. J Pediatr Orthop 2010; 30: 169–173. 20179565 10.1097/BPO.0b013e3181d07593

[86] Shore BJ McCarthy J Shrader MW , et al. Anterior distal femoral hemiepiphysiodesis in children with cerebral palsy: establishing surgical indications and techniques using the modified Delphi method and literature review. J Child Orthop 2022; 16: 65–74. 35615394 10.1177/18632521221087529 PMC9124914

[87] Halloum A Kold S Rölfing JD , et al. Correction of rotational deformities in long bones using guided growth: a scoping review. Efort Open Reviews 2024; 9: 119–128. 38308954 10.1530/EOR-23-0149 PMC10873243

[88] Paley D Shannon C. Rotational guided growth: a preliminary study of its use in children. Children (Basel) 2022; 10: 70. 36670621 10.3390/children10010070 PMC9856838

[89] Metaizeau JD Denis D Louis D. New femoral derotation technique based on guided growth in children. Orthop Traumatol Surg Res 2019; 105: 1175–1179. 31358462 10.1016/j.otsr.2019.06.005

[90] Halloum A Rahbek O Gholinezhad S , et al. A novel plate design for rotational guided growth: an experimental study in immature porcine femurs. J Orthop Res 2025; 43: 617–621. 39569605 10.1002/jor.26019 PMC11806649

[91] Arami A Bar-On E Herman A , et al. Guiding femoral rotational growth in an animal model. J Bone Joint Surg Am 2013; 95: 2022–2027. 24257660 10.2106/JBJS.L.00819

[92] Cobanoglu M Cullu E Kilimci FS , et al. Rotational deformities of the long bones can be corrected with rotationally guided growth during the growth phase. Acta Orthop 2016; 87: 301–305. 26900795 10.3109/17453674.2016.1152450 PMC4900079

[93] Lazarus DE Farnsworth CL Jeffords ME , et al. Torsional growth modulation of long bones by oblique plating in a rabbit model. J Pediatr Orthop 2018; 38: e97–e103. 10.1097/BPO.0000000000001106 29189535

[94] Zaidman M Simanovsky N Goldman V , et al. Correction of femoral torsional deformities by rotational guided growth. J Clin Med 2024; 13: 7514. 39768437 10.3390/jcm13247514 PMC11677953

[95] Abood AA Rolfing JD Halloum A , et al. An innovative plate concept for rotational guided growth: a porcine pilot study. Cureus 2024; 16: e58169. 10.7759/cureus.58169 PMC11015357 38616978

[96] Martel GA Holmes L Sobrado G , et al. Rotational-guided growth. J Limb Length Reconstr 2018; 4: 97–105.

[97] Noonan KJ Feinberg JR Levenda A , et al. Natural history of multiple hereditary osteochondromatosis of the lower extremity and ankle. J Pediatr Orthop 2002; 22: 120–124. 11744867

[98] Driscoll MD Linton J Sullivan E , et al. Medial malleolar screw versus tension-band plate hemiepiphysiodesis for ankle valgus in the skeletally immature. J Pediatr Orthop 2014; 34: 441–446. 24172668 10.1097/BPO.0000000000000116

[99] Stevens PM Kennedy JM Hung M. Guided growth for ankle valgus. J Pediatr Orthop 2011; 31: 878–883. 22101668 10.1097/BPO.0b013e318236b1df PMC3227545

[100] Chang FM Ma J Pan Z , et al. Rate of correction and recurrence of ankle valgus in children using a transphyseal medial malleolar screw. J Pediatr Orthop 2015; 35: 589–592. 26251960 10.1097/BPO.0000000000000333

[101] Davids JR Valadie AL Ferguson RL , et al. Surgical management of ankle valgus in children: use of a transphyseal medial malleolar screw. J Pediatr Orthop 1997; 17: 3–8. 8989691

[102] Laine JC Novotny SA Weber EW , et al. Distal tibial guided growth for anterolateral bowing of the tibia: fracture may be prevented. J Bone Joint Surg Am 2020; 102: 2077–2086. 33093298 10.2106/JBJS.20.00657

[103] Todderud JE Carlson SW Larson AN. Guided growth to treat anterolateral tibial bowing associated with congenital pseudarthrosis of the Tibia. J Pediatr Orthop 2024; 44: e560–e565. 10.1097/BPO.0000000000002683 38835290

[104] Al-Aubaidi Z Lundgaard B Pedersen NW. Anterior distal tibial epiphysiodesis for the treatment of recurrent equinus deformity after surgical treatment of clubfeet. J Pediatr Orthop 2011; 31: 716–720. 21841451 10.1097/BPO.0b013e31822109b6

[105] Ebert N Ballhause TM Babin K , et al. Correction of recurrent equinus deformity in surgically treated clubfeet by anterior distal tibial hemiepiphysiodesis. J Pediatr Orthop 2020; 40: 520–525. 32555046 10.1097/BPO.0000000000001609

[106] Zargarbashi R Abdi R Bozorgmanesh M , et al. Anterior distal hemiepiphysiodesis of tibia for treatment of recurrent equinus deformity due to flat-top talus in surgically treated clubfoot. J Foot Ankle Surg 2020; 59: 418–422. 32131014 10.1053/j.jfas.2019.08.018

[107] Besselaar AT. Walking with the clubfoot child. Maastricht University, 2025. www.orthopeden.org

[108] Al-Mohrej OA Ade-Conde AM Ade-Conde OS , et al. Hemiepiphysiodesis for juvenile hallux valgus deformity: a systematic review. Foot Ankle Surg 2023; 29: 448–454. 37419765 10.1016/j.fas.2023.06.010

[109] Sanpera I Jr Frontera-Juan G Sanpera-Iglesias J , et al. Innovative treatment for pes cavovarus: a pilot study of 13 children. Acta Orthop 2018; 89: 668–673. 29911919 10.1080/17453674.2018.1486525 PMC6300739

[110] Domingues LS Norte S Thusing M , et al. Is there a place for dorsal hemiepiphysiodesis of the first metatarsal in the treatment of pes cavovarus? J Pediatr Orthop B 2025; 34: 151–156. 39302844 10.1097/BPB.0000000000001209 PMC11776887

[111] Siemensma MF van Bergen CJA van Es EM , et al. Indications and timing of guided growth techniques for pediatric upper extremity deformities: a literature review. Children (Basel) 2023; 10: 195. 36832323 10.3390/children10020195 PMC9954695

[112] Soldado F Diaz-Gallardo P Cherqaoui A , et al. Unsuccessful mid-term results for distal humeral hemiepiphysiodesis to treat cubitus varus deformity in young children. J Pediatr Orthop B 2022; 31: 431–433. 35102055 10.1097/BPB.0000000000000950

[113] Martínez-Álvarez S Galán-Olleros M Alonso-Hernández J , et al. Guided growth for the treatment of cubitus varus in children: medium- to long-term results. J Clin Med 2023; 12: 2632. 37048715 10.3390/jcm12072632 PMC10095142

[114] Amnah Almahmudi DA Hussain Assaggaf . Lateral condyle temporary hemiepiphysiodesis in treatment of cubitus varus deformity post supracondylar humerus fracture: a case report. Med Case Rep 2020; 6: 150.

[115] Verka PS. A novel minimally invasive method in pediatric cubitus varus deformity, distal lateral humerus hemiepiphysiodesis: a case report. J Pediatr Neurol Disord 2021; 4: 1–2.

[116] Soler-Jimenez A Gonzalez-Herranz P Pensado-Senoris N. Guided growth with minifragment plates for angular deformities in the distal radius in skeletally immature patients. preliminary results. J Pediatr Orthop 2024; 44: e691–e697. 10.1097/BPO.0000000000002722 38767293

[117] Kelly JP James MA. Radiographic outcomes of hemiepiphyseal stapling for distal radius deformity due to multiple hereditary exostoses. J Pediatr Orthop 2016; 36: 42–47. 25633611 10.1097/BPO.0000000000000394

[118] Scheider P Ganger R Farr S. Temporary epiphysiodesis in adolescent patients with ulnocarpal impaction syndrome: a preliminary case series of seven wrists. J Pediatr Orthop B 2021; 30: 601–604. 32932414 10.1097/BPB.0000000000000805 PMC8480517

[119] Arora AS Chung KC Otto W. Madelung and the recognition of Madelung’s deformity. J Hand Surg Am 2006; 31: 177–182. 16473675 10.1016/j.jhsa.2005.09.001

[120] Farr S Kalish LA Bae DS , et al. Radiographic criteria for undergoing an ulnar shortening osteotomy in madelung deformity: a long-term experience from a single institution. J Pediatr Orthop 2016; 36: 310–315. 25757208 10.1097/BPO.0000000000000434

